# Can Teleworking Improve Workers’ Job Satisfaction? Exploring the Roles of Gender and Emotional Well-Being

**DOI:** 10.1007/s11482-023-10145-4

**Published:** 2023-02-04

**Authors:** Zhuofei Lu, Wei Zhuang

**Affiliations:** 1 Department of Social Statistics, University of Manchester, Oxford Road, M13 9PL Manchester, UK; 2 University of Manchester, Oxford Road, M13 9PL, Manchester, UK

**Keywords:** Enjoyment at work, Gender, Job satisfaction, Teleworking, Work autonomy

## Abstract

With the rise of teleworking during the past decades, the impacts of teleworking on job satisfaction have been extensively debated. Teleworking might benefit workers by improving work-life balance and emotional well-being, but it also brings considerable challenges. This study empirically investigates the impacts of teleworking on workers’ enjoyment across daily working episodes and job satisfaction and its gendered patterns, using Ordinary Least Squares regressions and the latest nationally representative time-use survey data in the UK. Moreover, it uses the Karlson/Holm/Breen (KHB) decomposition method to examine the role of enjoyment at work in mediating the associations between teleworking and job satisfaction. Overall, this study yields two major findings. First, among men, teleworkers tend to have higher levels of enjoyment at work and job satisfaction, but this is not the case for women. Second, around 46% of teleworking’s positive impacts on men’s job satisfaction can be explained by higher levels of enjoyment at work. Taken together, by integrating different theoretical perspectives on teleworking, gender and emotional well-being, this study provides interdisciplinary insights into the nuanced social consequences of teleworking, highlights the disadvantaged position of women in the use of teleworking, and demonstrates the need to enhance emotional well-being in future labour market policies.

## Introduction

Teleworking refers to the practice of workers working outside of the traditional single workplace, or while on the move (Morganson et al., 2010). Organisations and governments had actively promoted teleworking over the past decades, especially during the Covid-19 pandemic when it was advocated to prevent the spread of the disease and social issues (Li & Wang, 2020; Pataki-Bittó & Kun, 2022). However, there remain uncertainties about whether teleworking promotes or damages workers’ job satisfaction and enjoyment at work, and there has yet to be empirical research on its intersection with gender or its underlying emotional well-being mechanism.

In addition to the lack of empirical research, the existing theoretical arguments and findings about the impact of teleworking on job satisfaction and its gendered patterns are conflicting. On the one hand, the Job Demand Control (JDC) model (Karasek, 1979; Wheatley, 2012) predicts that workers, especially female workers, will have more work autonomy to juggle work and family commitments when using teleworking and will thereby have better emotional well-being and more job satisfaction. For instance, a stream of studies has found that workers report higher job satisfaction when using teleworking, while such impacts are more pronounced among women (Coron, 2022; Wheatley, 2012). On the other hand, the role blurring theory (Desrochers & Sargent, 2004) predicts that teleworking can blur the boundaries between workers’ occupational commitments and private lives, thereby reducing their job satisfaction and harming their work-life balance. For example, studies find that teleworking increases workers’ subjective time pressure and irritability, especially among men (Pataki-Bittó & Kun, 2022; Song & Gao, 2019). Given the inconsistent theoretical predictions and findings, the first objective of the study is to investigate the impacts of teleworking on workers’ emotional well-being at work and overall job satisfaction while considering the potential moderating role of gender.

Although some scholars have emphasised the importance of workers’ instantaneous emotional well-being (i.e., enjoyment at work) in predicting job satisfaction (Brief & Weiss, 2002; Wegge et al., 2006a), the investigation of workers’ enjoyment at work is rather absent in most studies on the association between teleworking and job satisfaction (Morganson et al., 2010; Pataki-Bittó & Kun, 2022; Teo & Lim, 1998; Wheatley, 2012). Failing to consider underlying factors like instantaneous enjoyment at work prevents us from gaining a nuanced understanding of the connection between teleworking and job satisfaction (Brief & Weiss, 2002; Wegge et al., 2006a). Even though there is a stream of studies that has investigated the associations between teleworking and emotional well-being (Anderson et al., 2015; Vega et al., 2015) and the underlying emotional well-being mechanism at the organisational level by using stylised survey data, there is selection bias, memory bias and estimate inaccuracy in their analyses (Kan & Pudney, 2008). Moreover, the potential gender differences in these underlying factors remain poorly researched with time diary evidence. Thus, drawing on the previous literature, this study’s second objective is to use the Karlson/Holm/Breen (KHB) decomposition method to investigate the role of emotional well-being at work in mediating the impacts of teleworking on the job satisfaction of workers of different genders.

By achieving both objectives, this study makes three important contributions to the literature. First, we empirically extend the current literature by analysing the impacts of teleworking on workers’ instantaneous enjoyment at work and job satisfaction. Operationally, this study uses 24-hour time use data to measure workers’ instantaneous emotion (enjoyment) during each work episode. Secondly, we provide novel insights into the gender differences in the impacts of teleworking. Thirdly, we bridge the divergent theoretical understandings of teleworking, gender, emotional well-being and job satisfaction by analysing the role of enjoyment at work in mediating the associations between teleworking and job satisfaction.

### Teleworking, Subjective Well-Being and Gender

Although governments and organisations promoted teleworking to enhance job performance and work-life balance around the world during the Covid-19 pandemic, whether teleworking improves job satisfaction and other subjective well-being outcomes is still subject to extensive debates. The theoretical predictions and empirical findings about teleworkers’ job satisfaction and enjoyment at work are conflicting. In addition, gender inequalities in the household and labour market still remain, with women spending more time on housework and childcare and less time in full-time work than men (M.-Y. Kan & Laurie, 2018). Thus, the consequence of teleworking might differ by gender.

On the one hand, some scholars argue that teleworking can provide workers with more job autonomy and flexibility, thereby reducing work-related stress and improving subjective well-being. Specifically, the Job Demand Control (JDC) model (Karasek, 1979) indicates that high degrees of work autonomy can promote workers’ job performance and satisfaction by alleviating work pressure, reducing workloads and ensuring job satisfaction (Grönlund, 2007; Karasek, 1979; Wheatley, 2017). Work autonomy and flexibility are important in addressing mental issues during and after workers’ working events (Chung, 2017; Lopes et al., 2014). Thus, workers may have less work-related stress during teleworking since they are spatially and psychologically removed from “direct, face-to-face supervision” with higher levels of work autonomy (Fonner & Roloff, 2010). For women, in particular, there is a rich research tradition investigating how teleworking (especially working from home) benefits subjective well-being by providing more work autonomy and flexibility to balance occupation commitments and domestic responsibilities (Chung, 2022; Chung & van der Horst, 2018). Indeed, many studies have found that the benefits of teleworking on subjective well-being are more pronounced among women than men (Bae & Kim, 2016; Wheatley, 2012) since they have a higher demand for work autonomy and flexibility to alleviate work-family conflicts (Glavin & Schieman, 2012; Li & Wang, 2022; Wang & Lu, 2022). Nonetheless, although men may benefit less from teleworking than women in terms of alleviating work-family conflicts, teleworking can benefit male workers in other ways by alleviating the mental strain brought on by high job demand. This is because traditional gender norms and occupational ethics expect men to do more ‘active jobs’, which are highly demanding but with better prospects and payments (Karasek, 1979; West & Zimmerman, 1987). For instance, previous research on the career expectations of workers found that women tend to prioritise their ‘work-life balance’, while men prioritise a ‘high salary’ or ‘prospects’ (Schweitzer et al., 2011, 2014). Thus, male taleworkers may have better enjoyment since they have more work autonomy to alleviate their relatively higher work demand. Therefore, drawing on the theoretical perspectives from the JDC model and the sociology of gender, teleworking might benefit both men and women but through different mechanisms.

On the other hand, another stream of studies challenges the assumptions of the JDC model, and indicates that teleworking may reduce workers’ level of enjoyment at work and job satisfaction. Specifically, the role blurring theory (Desrochers & Sargent, 2004) indicates that working out of the single traditional workplace, in particular working at home, will blur the boundary between job commitments and family responsibilities, thereby increasing a series of adverse effects. Such role-blurring patterns can influence workers’ working events during teleworking, for instance, by increasing the frequency of multitasking and fragmented working episodes (Cornwell, 2013; Offer & Schneider, 2011). Women are particularly vulnerable to role blurring since they are more likely to use teleworking to facilitate family demands (Abendroth, 2022; Kim et al., 2019). For example, previous studies have found that women working from home tend to suffer more interruptions by household needs and have a more fragmented time schedule (Powell & Craig, 2015), which means they will suffer low time quality and enjoyment when teleworking (Craig & Brown, 2017). Moreover, the ‘flexibility paradox’ thesis indicates that work autonomy might not always facilitate work-family balance but instead lead to longer paid and unpaid working hours (Chung, 2022). A strand of the latest empirical evidence indicates that women are more likely to have more multitasking episodes, work-family conflicts and longer total (paid and unpaid) working hours (Chung & Booker, 2022; Yucel & Chung, 2021), leading to worse emotional well-being and job satisfaction status. It is also worth noting that female workers’ disadvantaged position in the labour market places a “glass ceiling” (Clawson, 2014; England et al., 2020; Wang, Zixin, et al., 2022) on their opportunities for promotion into higher occupational positions that offer more opportunities to do telework. This means that women may have difficulty using teleworking because they are concentrated in lower occupational positions with less job quality but more demand from households. Meanwhile, men are more likely to prioritise and identify with their work commitments, with teleworking arrangements resulting in more overtime (Chung & van der Horst, 2020), and so face a form of the ‘flexibility paradox’. In addition, teleworkers might suffer from worse prospects and feelings of being marginalised (Tietze & Musson, 2010; Williams et al., 2013) due to the lack of communication with employers and colleagues, while such adverse effects might be more pronounced among men due to restrictions from traditional gender norms (Chung & van der Horst, 2020; Kim et al., 2019; West & Zimmerman, 1987).

Overall, previous research has conflicting predictions about the gendered associations between teleworking and workers’ subjective well-being (i.e., work-life balance, job satisfaction, time quality and mental strain). Therefore, the empirical analysis in this paper seeks to answer the following research question: How does teleworking shape workers’ enjoyment at work and job satisfaction across gender?

### The Underlying Emotional Well-Being Mechanisms

In this section, we explore the underlying emotional well-being mechanisms. Generally, job satisfaction consists of two components: (1) the cognitive dimension, represented by the evaluative judgement about a period of work experience, and (2) the emotional well-being dimension, represented by one’s instantaneous feeling of worktime flow (Brief & Weiss, 2002; Judge et al., 2001; Veenhoven & Publications, 2008). The previous studies on emotional well-being, job satisfaction and work environment have made concerted efforts to explain how work environments shape workers’ feelings about different work-related events, thereby influencing job satisfaction (Brief & Weiss, 2002; Cernas-Ortiz & Wai-Kwan, 2021). However, most of the studies on teleworking and job satisfaction generally continue to focus loosely on the cognitive dimension while ignoring workers’ emotional well-being. Failing to consider the role of emotional well-being in the impacts of teleworking prevents us from gaining a nuanced understanding of teleworking and job satisfaction (Brief & Weiss, 2002). Even though there is a strand of studies that has investigated the associations between teleworking and emotional well-being (Anderson et al., 2015; Vega et al., 2015), they collect respondents’ emotional well-being by using stylised survey questions instead of using time diaries. This can generate memory bias and estimate inaccuracy during the analyses (Kan & Pudney, 2008), methodologically speaking. Many scholars in the field of sociology and economics have emphasised the advantages of using the time-diary method or some other similar approaches in collecting and measuring respondents’ emotional well-being (Hoang & Knabe, 2020; Zuzanek & Zuzanek, 2014). Therefore, we use time diary data to measure workers’ instantaneous emotions at work to capture their emotional well-being.

In this study, we assume that teleworking can influence not only workers’ enjoyment at work but also job satisfaction, and the associations between teleworking and job satisfaction can be mediated by workers’ enjoyment at work. This is because the predictions from the JDC model and the role blurring theory can be embedded within the framework of the affective events theory (AET). In the first place, according to Weiss and Cropanzano’s (1996) affective events theory (AET), events are the causes of workers’ emotions, and the raw elements that combine to generate the emotional components of job satisfaction are mood and emotions experienced while working. Thus, how workers feel at work is led by the events that happen in the workplace, and the feelings at work further affect workers’ satisfaction with their job (Weiss & Cropanzano, 1996). Many studies have empirically demonstrated that workers with more enjoyment at work tend to have higher levels of overall job satisfaction (e.g., Fisher 2002; Wegge et al., 2006; Weiss et al., 1999). In the second place, as mentioned in Sect. 1.1, the predictions from the JDC model and the role blurring theory both suggest that teleworking can influence workers’ way of experiencing work-related events (e.g., with more flexibility or more role blurring). For instance, the JDC model predicts that teleworkers with more work autonomy can have better enjoyment during the working episodes since they might feel better job quality and work-life balance (Anderson et al., 2015). By contrast, the role blurring theory assumes that teleworkers, especially women homeworkers, might suffer less enjoyment during working episodes since they have higher risks of multitasking and temporal interruptions by family demands (Anderson et al., 2015). These two strands of predictions can also be found in AET but with less explanation of the mechanism (Anderson et al., 2015). The arguments from the JDC model and the role blurring theory contribute supplementary theoretical explanations to AET’s framework. Therefore, it is reasonable to assume that if teleworking can influence workers’ enjoyment at work and job satisfaction by bringing more work flexibility or role blurring/conflicts, there might be underlying emotional well-being mechanisms behind the associations.

Moreover, the role of enjoyment at work in mediating the positive impacts of teleworking on job satisfaction might also vary by gender. As mentioned above, gender inequality in the division of housework and labour participation may moderate the impacts of teleworking on enjoyment at work and job satisfaction. For women, the increased enjoyment at work might significantly increase the positive impacts of teleworking on job satisfaction by buffering the adverse effects of work-family conflicts. On the other hand, the benefits of teleworking might be offset by increased multitasking and more hours of unpaid work. Meanwhile, for men, the increased enjoyment at work might significantly mediate the positive impacts of teleworking on job satisfaction by buffering the mental strain brought by high job demand. On the other hand, such benefits might not be strong enough and then offset by worse prospects and feelings of being marginalised. Therefore, the study’s second research question is: Does workers’ enjoyment at work mediate the positive impacts of teleworking on job satisfaction, (if so) whether there are gender differences in the relationships?

## Methods

### Data

This study uses the data from the UK Time-Use Survey (UKTUS) 2014/2015, the latest nationally representative time-use survey in the UK, to include detailed information about respondents’ workplace arrangements and instantaneous feelings during the day. The original sample includes 9388 individuals from 4239 households (Hoang & Knabe, 2020). The UKTUS sampled around 11,000 eligible households drawn from the Postcode Address File (PAF) system by using a multi-stage stratified probability sampling design. During the survey, respondents were required to record their main and secondary activities and level of enjoyment across 144 10-minute episodes throughout a weekday and a weekend day. In addition, the respondents were also asked to attend an interview after completing the diary, which collected substantive information about their socioeconomic information and subjective well-being status. This study focuses on the subset of adults who reported working in paid employment. Moreover, the study excluded the diaries for weekends, and non-work days. After excluding the samples with missing data, the study’s sample comprises 931 workers who completely recorded their activities and enjoyment across each episode on a typical weekday. More details about the analytic sample can be seen in Table 1.

**Table 1:** Weighted sample descriptive statistics

	Non-teleworkers		Teleworkers	
Variables	%, Mean	SD	%, Mean	SD
Enjoyment at work	4.59	1.31	4.75	1.17
Job satisfaction	5.06	1.57	5.41	1.39
Age	40	12.9	43	11.9
Sex				
Male	47%		66%	
Female	53%		34%	
The occupational class Large employers and higher managerial	2%		5%	
Higher professional	12%		20%	
Lower managerial and professional	28%		26%	
Intermediate	21%		12%	
Small employers & own account workers	0%		3%	
Lower supervisory and technical	5%		5%	
Semi-routine	20%		14%	
Routine & manual	12%		15%	
The presence of long-standing illness				
Yes	22%		31%	
No	78%		69%	
The presence of children under 16				
Yes	62%		64%	
No	38%		36%	
General health status				
Very good	39%		38%	
Good	46%		44%	
Fair	14%		17%	
Bad	1%		1%	
Very bad	0%		0%	
Paid work (hours per day)	8.49	2.43	9.10	2.59
Routine work (hours per day)	0.89	0.92	0.69	0.77
Childcare (hours per day)	0.36	0.79	0.29	0.85
Logged household income	7.92	0.76	8.11	0.85
Number of respondents (N = 929)	N = 683 (73%)		N = 246 (27%)	

Note: % = Proportion, M = Mean, SD = Standard deviation

### Measurements

**Enjoyment at work** is measured by respondents’ average enjoyment score of their work-related activities in a typical workday. Specifically, during the survey, respondents were asked to rate their enjoyment of each 10 min episode. The diary’s specific question of ‘enjoyment’ is “how much did you enjoy this time slot?”, with the answers ranging from 1 (not at all) to 7 (very much). We begin with identifying workers’ work-related activities within a typical workday. Work activities encompass the activities related to respondents’ primary and secondary jobs, including working, work-related travelling, work-related meetings and other unspecified working-related activities. Then, we calculated the average enjoyment score of all work-related activities within the day and generated a continuous variable, ‘enjoyment at work’, ranging from 1 (not at all) to 7 (very much). **Job satisfaction** is measured by respondents’ answers to the question “what is the level of satisfaction with your job?” ranging from 1 (completely dissatisfied) to 7 (completely satisfied). Workers’ use of **teleworking** is measured by respondents’ answers to the interview questions about where they mainly work, including (1) mainly working at a single traditional workplace (e.g., office or factory), (2) mainly working at home, (3) mainly working in a variety of different places of work, (4) mainly working on the move (e.g., delivering products or driving). The study dichotomises the answers to (1) teleworking: those mainly working at home, in a variety of different places of work, or on the move; and (2) no teleworking: those who mainly work at a single traditional workplace, such as traditional office or factory.

The study also controls for a series of socioeconomic characteristics that previous studies have identified (Athey et al., 2016; Hofmans et al., 2014; Lee & Jang, 2020; Sanz-Vergel & Rodríguez-Muñoz, 2013) to correlate with enjoyment at work and job satisfaction, including gender, occupational class, logged household monthly income, the presence of long-term illnesses, the presence of children under 16, general health status and respondents’ time (hours per day) spent on paid work, routine housework and childcare. Occupational class is measured by the eight-category version of the National Statistics Socio-economic Classification (NS-SEC), which was developed from a widely used and reliable measurement of social class, known as the Goldthorpe Schema (Erikson & Goldthorpe, 2010).

### Analytical Strategies

This study presents a series of descriptive analyses to show the sample details. In line with previous studies (Powell & Craig, 2015; Yucel & Chung, 2021), this study uses Ordinary Least Squares regressions to examine the relationship between teleworking, workers’ enjoyment at work and job satisfaction. Interaction terms are included in the regressions to test the moderating role of gender. All the regressions use the weight suggested in the dataset to adjust the unequal sampling fraction. All of the study’s models have passed the tests for multicollinearity by examining the variance inflation factor (the VIF scores of all the variables in the models are smaller than 1.5). In addition, the study adopts the Karlson/Holm/Breen (KHB) method (Breen et al., 2013; Kohler et al., 2011) to investigate the potential mediating role of enjoyment at work in the associations between teleworking and job satisfaction. The KHB method decomposes the total effect of the variable into direct effects and indirect effects (Wang et al., 2021). In addition, the KHB method also calculates the proportion of the main association explained by the mediator.

## Results

**Fig. 1:**
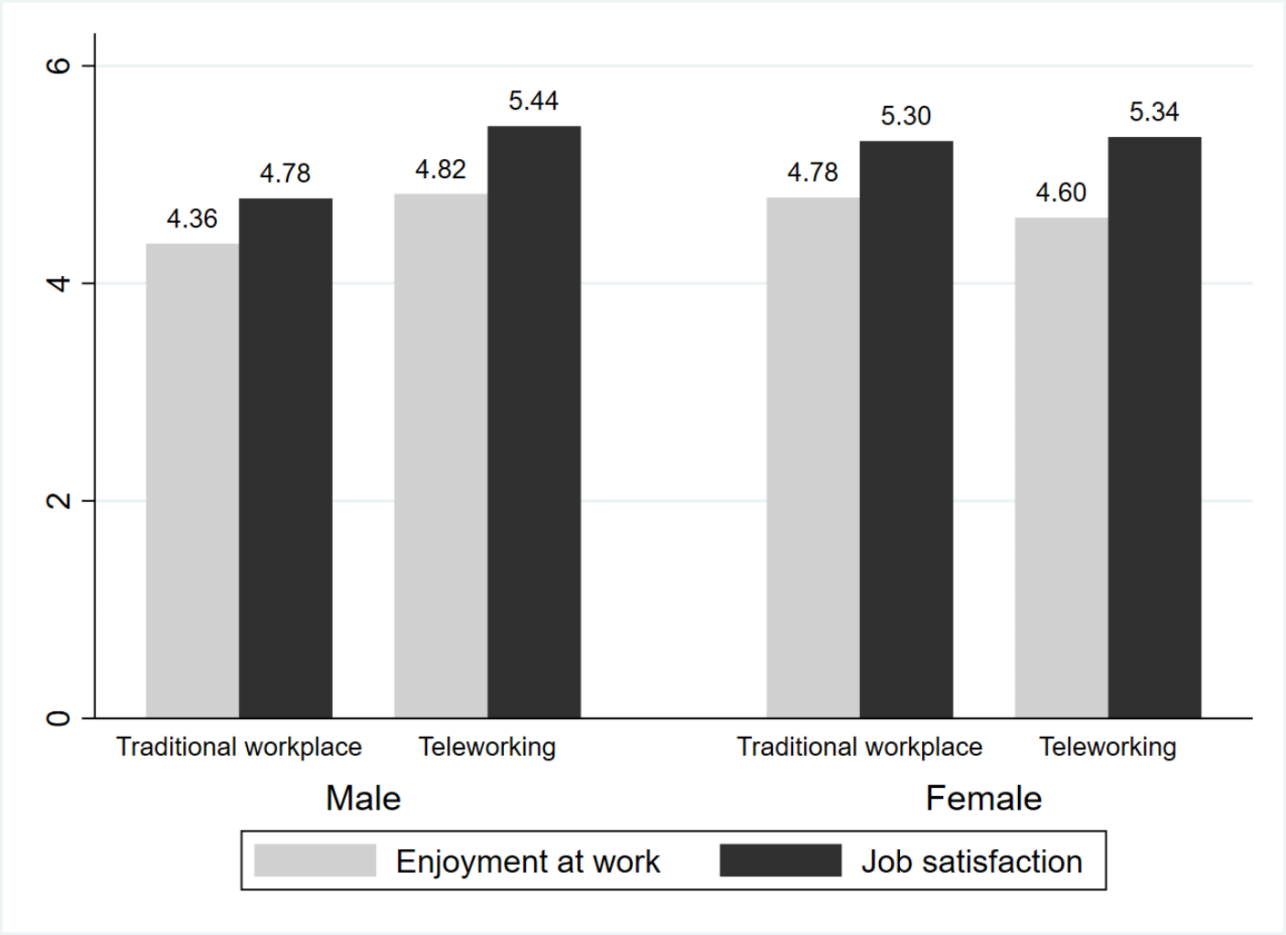
The weighted mean of workers’ enjoyment at work and job satisfaction of teleworkers and non-teleworkers (by gender)

Table 1 shows the results of the weighted sample descriptive analyses. The final analytic sample includes 683 (73%) non-teleworkers and 246 (27%) teleworkers. Teleworkers and non-teleworkers have different demographic and socioeconomic characteristics. For instance, women make up a larger proportion of non-teleworkers than men, while men are the majority of teleworkers and account for 32% more than women. In addition, teleworkers are slightly more likely to report long-standing illnesses and the presence of children. Teleworkers generally report higher income and longer paid working hours but shorter routine and childcare hours. It is worth noting that there is no participant who reports a ‘very bad’ general health status and no self-employed who are not teleworkers. During the data cleaning, we only kept the typical workday diaries (without sick leave diaries). This treatment can exclude most workers who have a ‘very bad’ health status. In addition, all of the ‘small employers/own account workers’ are teleworkers since they have more flexibility to decide where to work. Table 1 also presents the weighted mean values of enjoyment at work and job satisfaction. As shown in Tables 1, teleworkers have a higher mean enjoyment at work (4.75 versus 4.59) and job satisfaction (5.41 versus 5.06) than non-teleworkers. Furthermore, Fig. 1 plots the mean values of enjoyment at work and job satisfaction of teleworkers and non-teleworkers across gender. As for men, teleworkers report higher mean enjoyment at work (4.82 versus 4.36) and job satisfaction (5.44 versus 4.78) than non-teleworkers. By contrast, as for women, teleworkers report lower mean enjoyment at work than non-teleworkers (4.60 versus 4.78). The mean of women teleworkers’ job satisfaction is slightly higher than non-teleworkers (5.34 versus 5.30). Given these demographic and socioeconomic differences, it is crucial to take these factors into account in the multivariate regression analyses.

Table 2 presents the results of a series of multivariate linear regressions predicting the impacts of teleworking on the workers’ enjoyment at work and job satisfaction over a weekday. In particular, Model 1 indicates that those who used teleworking tend to report higher levels of enjoyment at work than non-teleworkers (coefficient = 0.24, SE = 0.10, p < 0.05). Also, Model 2 in Table 2 suggests that those who used teleworking tend to report higher job satisfaction than non-teleworkers (coefficient = 0.44, SE = 0.12, p < 0.001). Moreover, gender is significant in Model 2, with women generally reporting more job satisfaction than men. Thus, the results in Table 2 generally indicate that teleworking has significantly associated with better enjoyment at work and job satisfaction.

**Table 2:** Ordinary Least Squares (OLS) regressions predicting the impacts of teleworking on the workers’ enjoyment at work and job satisfaction over a weekday

	Enjoyment at work	Job satisfaction
	Model 1	Model 2
Teleworking (Ref.= No)		
Yes	0.24*	0.44***
	(0.10)	(0.12)
Gender (Ref.= Male)		
Female	0.15	0.32**
	(0.10)	(0.12)
Constant	4.06***	4.32***
	(0.63)	(0.79)
Observations	929	929
R-squared	0.05	0.06

**Note**: Standard errors are in parentheses. *** p < 0.001, ** p < 0.01, * p < 0.05. All models control for occupational class, logged household monthly income, long-term illnesses, children under 16, general health status and respondents’ time spent on paid work, routine housework and childcare. See Table A1 in the appendix for full details about the coefficients of the covariates

**Fig. 2:**
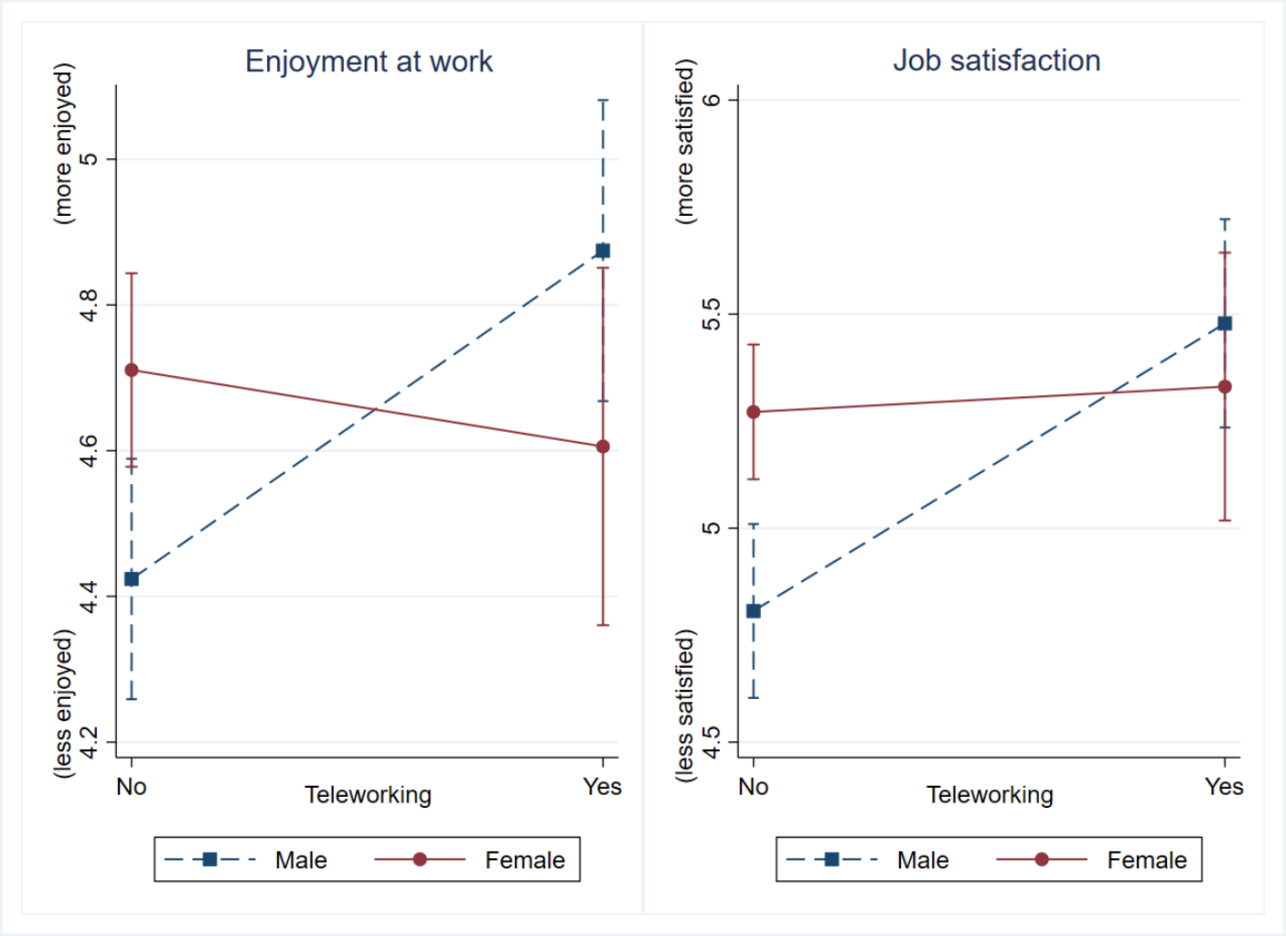
The interactions between teleworking and gender

Next, the study tests the role of gender. As shown in Table 3, the interactions between gender and teleworking are significant in the associations between teleworking and enjoyment at work (coefficient = -0.56, SE = 0.19, p < 0.01) and the associations between teleworking and job satisfaction (coefficient = -0.61, SE = 0.24, p < 0.01), confirming the moderating role of gender. Figure 2 plots the interactions between teleworking and gender. As shown on the left side of Fig. 2, the impacts of teleworking on enjoyment at work are positive among men (the gradient of the line with the square tag is upward), but negative among women (the gradient of the line with the circle tag is downward). Regarding job satisfaction, as shown on the right side of Fig. 2, male teleworkers report significantly higher levels of job satisfaction than non-teleworkers. By contrast, amongst women, levels of job satisfaction are generally similar between teleworkers and non-teleworkers. We conducted further robustness checks by analysing the impact of teleworking on enjoyment at work and job satisfaction within gendered samples. The results of the robustness checks are generally consistent with the results of the interaction tests (see Table A2 in the appendix), with teleworking can only be significantly associated with men’s enjoyment at work and job satisfaction but not women’s. Taken together, the study finds that gender can significantly moderate the impacts of teleworking on workers’ enjoyment at work and job satisfaction, answering the study’s first research question. Men can benefit from teleworking in terms of both enjoyment at work and job satisfaction, but not women.

**Table 3:** Ordinary Least Squares (OLS) regressions predicting the moderation impacts of gender in the impacts of teleworking on the workers’ job satisfaction

	Enjoyment at work	Job satisfaction
	Model 1	Model 2
Teleworking (Ref.= No)		
Yes	0.45***	0.67***
	(0.13)	(0.16)
Gender (Ref.= Male)		
Female	0.29***	0.47***
	(0.11)	(0.13)
Teleworking × Gender (Ref.= Male)		
Yes × Female	-0.56**	-0.61**
	**(0.19)**	**(0.24)**
Constant	3.93***	4.17***
	(0.63)	(0.77)
Observations	929	929
R-squared	0.06	0.07

**Note**: Standard errors are in parentheses. *** p < 0.001, ** p < 0.01, * p < 0.05. All models control for occupational class, logged household monthly income, long-term illnesses, children under 16, general health status and respondents’ time spent on paid work, routine housework and childcare

Finally, the study examines the potential mediating role of enjoyment at work. Table 4 illustrates the adjusted results of the mediation analysis by using the KHB decomposition method. As shown in panel A (total sample) of Table 4, the total and direct effects of teleworking on workers’ job satisfaction are 0.43 (p < 0.001) and 0.29 (p < 0.01), respectively. The indirect effect via enjoyment at work is 0.13 (p < 0.05), with 30% of the total effect being mediated. Next, we repeat the mediation tests with gendered samples. As shown in panel B (men) of Table 4, the total and direct effects of teleworking on workers’ job satisfaction are 0.67 (p < 0.001) and 0.36 (p < 0.01). The indirect effect via enjoyment at work is 0.31 (p < 0.001), with 46% of the total effect being mediated, which is more pronounced than among the total sample. By contrast, as shown in panel C (women), the total and direct effects of teleworking on workers’ job satisfaction are both insignificant. No significant indirect effect via enjoyment at work was observed among women. Overall, the results of the mediation analysis answer the study’s second research question. Enjoyment at work can mediate the impacts of teleworking on job satisfaction, while such a mediating role is only significant among men.

**Table 4:** KHB decomposition method examining the mediation effects of enjoyment at work

Job satisfaction		Coefficient	95%CI	P value	Mediation (%)
Panel A: Total sample (N = 931); R-squared = 0.29					
Teleworking					
Total		0.43 (0.10)	0.23–0.63	< 0.001	
Direct		0.30 (0.10)	0.09–0.49	< 0.01	
Indirect via enjoyment at work		0.13 (0.06)	0.02–0.25	< 0.05	30%
Panel B: Male workers (N = 453); R-squared = 0.38					
Teleworking					
Total		0.67 (0.13)	0.41–0.93	< 0.001	
Direct		0.36 (0.13)	0.09–0.62	< 0.01	
Indirect via enjoyment at work		0.31 (0.09)	0.14–0.49	< 0.001	46%
Panel C: Female workers (N = 476); R-squared = 0.26					
Teleworking					
Total		0.24 (0.16)	-0.08-0.55	> 0.1	
Direct		0.25 (0.16)	-0.07-0.56	> 0.1	
Indirect via enjoyment at work		-0.01 (0.08)	-0.16-0.15	> 0.1	

Note: Standard errors in parentheses, CI = confidence interval. All models control for occupational class, logged household monthly income, long-term illnesses, children under 16, general health status and respondents’ time spent on paid work, routine housework and childcare

## Discussion and Conclusions

Over the past decades, the rise of teleworking has stimulated extensive debates over whether teleworking promotes workers’ emotional well-being and job satisfaction. Using nationally representative time diary data of 929 British workers and a series of statistical techniques (i.e., OLS regressions and the KHB method), this study examined whether teleworking influences workers’ enjoyment at work and job satisfaction, and if so, whether such associations varied by gender. In addition, this study examined the underlying emotional well-being mechanisms and the gender differences behind the associations between teleworking and job satisfaction, which is rarely discussed in previous studies on teleworking and job satisfaction. Overall, this study has yielded the following important findings.

First, the study finds that teleworkers tend to have better enjoyment at work and job satisfaction than non-teleworkers among men. This finding partially supports the arguments from the JDC model (Karasek, 1979) and the AET, which predicts that the high work autonomy brought by teleworking benefits workers’ emotional well-being and job satisfaction by alleviating mental strain and high demand. Regarding the gender differences, this study finds that women tend to be less likely to do teleworking than men, and the positive association between teleworking and the outcomes (job satisfaction and enjoyment at work) are not significant among women. This finding mirrors the predictions of the role-blurring theory (Desrochers & Sargent, 2004). Specifically, it shows that teleworking will lead to more multitasking and housework, thereby offsetting the benefits of teleworking. This offset pattern is pronounced among female workers due to their relatively disadvantaged position in both the labour markets and households (Glavin & Schieman, 2012; Wang & Li, 2022). By contrast, the predictions that teleworking would lead to ‘flexibility paradox’ issues (i.e., overtime), worse prospects, and feelings of being marginalised (Chung & van der Horst, 2020; Tietze & Musson, 2010) among men are not supported by the findings.

Second, the study, for the first time, finds that, among men, the association between teleworking and job satisfaction are mediated by their enjoyment at work. This finding bridges the research on teleworking, gender and job satisfaction through the lens of emotional well-being. It suggests the predictions of the Affective Events Theory (Wegge et al., 2006a), indicating that teleworking may improve men’s job satisfaction by improving their enjoyment at work. The theoretical perspectives on gender and work flexibility suggest that male workers are more likely to suffer high job demands and mental strain (Glavin & Schieman, 2012; Karasek, 1979; Schieman et al., 2006) but are less likely to use work autonomy to facilitate work-family balance during teleworking (Chung, 2017). Thus, as for men, the benefits of teleworking in alleviating work stress and mental strain might not be offset by increased unpaid work and multitasking. In contrast, previous studies that ignored the role of emotional well-being and its relationship with gender could overestimate or underestimate the impacts of teleworking on job satisfaction across gender.

This study has some limitations, which could point to potential directions for future studies. The first limitation of the study is that we cannot make causal inferences with the cross-sectional design. However, reverse causality is less likely because we do not find enough evidence from the current literature to suggest the impacts of job satisfaction on teleworking. Secondly, given that we are not able to identify the causal relationship between teleworking and job satisfaction, our mediation analysis of emotional well-being is also not causal. Thirdly, the study only explores workers’ teleworking and enjoyment at work on weekdays, which are relatively stable and predictable. In addition, the study does not analyse workers’ positive and negative emotions separately since the dataset does not contain that information. Future research could examine the emotional well-being of teleworkers at weekends or holidays and differentiate positive and negative emotions. Fourthly, this study focuses on the impacts of teleworking on individual-level subjective well-being. However, a growing body of studies highlights the linked lives between different family members within households (e.g., dual-earner families) (Inanc, 2018; Wang, Ling, et al., 2022). Thus, future research could examine the spillover effects of teleworking on enjoyment at work and job satisfaction across different family members (Kim et al., 2019; Wunder & Heineck, 2013), which could further advance our understanding of the social consequences of teleworking. Finally, although the weighted sample in the study can closely represent the national working population, it is unbalanced in terms of teleworking and gender. Future studies can use the upcoming wave of UKTUS to repeat the analyses with a larger sample size or use different weighting strategies.

These limitations should not, however, overshadow this study’s novel contributions to our understanding of the consequences of teleworking on job satisfaction across gender and its poorly understood underlying mechanisms involving enjoyment at work. One implication to note from this study is how increases in teleworking may exacerbate the existing gender inequality in households and the labour market in the UK. Although there has recently been an increased proportion of women using teleworking, especially during the Covid-19 pandemic (Chung et al., 2021; Xue & McMunn, 2021; Yucel & Chung, 2021), this study finds that women will not benefit from teleworking in terms of enjoyment at work and job satisfaction. Based on this, a way to produce greater gender equality in the labour market may be for policymakers to, on the one hand, keep ensuring male workers’ rights to use teleworking, on the other hand, promote more work-family balance interference to ensure the benefits of female teleworkers. Furthermore, future studies should also consider workers’ enjoyment at work as a crucial indicator of job satisfaction and be aware of its role in mediating the impacts of teleworking on job satisfaction.

## Data Availability

Data is available from an open-access public depository (UK Data Service); see more details from: https://www.timeuse.org/node/10833.

## Appendix

**Table A1:** Ordinary Least Squares (OLS) regressions predicting the impacts of teleworking on the workers’ enjoyment at work and job satisfaction over a weekday with coefficients of control variables

	Enjoyment at work	Job satisfaction	
	Model 1		Model 2
Teleworking (Ref.= No)			
Yes	0.24*		0.44***
	(0.10)		(0.12)
Gender (Ref.= Male)			
Female	0.15		0.32**
	(0.10)		(0.12)
Occupational class (Ref.= Large employers and higher managerial)			
Higher professional	0.24		0.43
	(0.24)		(0.38)
Lower managerial and professional	0.49*		0.44
	(0.22)		(0.37)
Intermediate	0.37		0.35
	(0.23)		(0.38)
Small employers and own account workers	0.96*		0.81
	(0.42)		(0.55)
Lower supervisory and technical	0.14		0.10
	(0.27)		(0.44)
Semi-routine	0.36		0.20
	(0.23)		(0.39)
Routine & manual	0.36		0.39
	(0.23)		(0.39)
Age	0.01 (0.00)		0.00 (0.00)
The presence of children under 16			
Yes	0.00		0.15
	(0.10)		(0.12)
The presence of longterm illnesses			
Yes	0.12		0.02
	(0.11)		(0.13)
Log household income (monthly)	-0.01		0.05
	(0.06)		(0.07)
General health status (Ref.= Very Good)			
Good	0.11		0.02
	(0.09)		(0.11)
Fair	-0.20		-0.51**
	(0.15)		(0.19)
Bad	-0.21		-1.11
	(0.28)		(0.61)
Very bad	-1.41***		0.16
	(0.20)		(0.22)
Paid work (hours per day)	-0.03		-0.04
	(0.02)		(0.03)
Routine-work time (hours per day)	0.12*		0.10
	(0.06)		(0.07)
Childcare time (hours per day)	-0.02		-0.04
	(0.06)		(0.07)
Constant	4.06***		4.32***
	(0.63)		(0.79)
Observations	929		929
R-squared	0.05		0.06

**Note**: Standard errors are in parentheses. *** p < 0.001, ** p < 0.01, * p < 0.05

**Table A2:** Ordinary Least Squares (OLS) regressions predicting the impacts of teleworking on the workers’ enjoyment at work and job satisfaction over a weekday (by gender)

	Enjoyment at work Job satisfaction			
	Males	Females	Males	Females
	Model 1	Model 2	Model 3	Model 4
Teleworking (Ref.= No)				
Yes	**0.51*****	-0.07	**0.70*****	0.19
	(0.14)	(0.14)	(0.16)	(0.18)
Constant	4.54***	3.92***	3.88***	5.38***
	(0.78)	(0.94)	(0.98)	(0.92)
Observations	453	476	453	476
R-squared	0.07	0.10	0.11	0.07

**Note**: Standard errors are in parentheses. *** p < 0.001, ** p < 0.01, * p < 0.05. All models control for occupational class, logged household monthly income, long-term illnesses, children under 16, general health status and respondents’ time spent on paid work, routine housework and childcare

## References

[CR1] Abendroth AK (2022). Transitions to parenthood, flexible working and time-based work-to-family conflicts: a gendered life course and organisational change perspective. Journal of Family Research.

[CR2] Anderson, A. J., Kaplan, S. A., & Vega, R. P. (2015). The impact of telework on emotional experience: when, and for whom, does telework improve daily affective well-being? *European Journal of Work and Organizational Psychology*, *24*(6), 10.1080/1359432X.2014.966086.

[CR3] Athey EK, Leslie MS, Briggs LA, Park J, Falk NL, Pericak A, El-Banna MM, Greene J (2016). How important are autonomy and work setting to nurse practitioners’ job satisfaction? [Article]. Journal of the American Association of Nurse Practitioners.

[CR4] Bae, K. bin, & Kim, D. (2016). The Impact of Decoupling of Telework on Job Satisfaction in U.S. Federal Agencies: Does Gender Matter? American Review of Public Administration, 46(3). 10.1177/0275074016637183

[CR5] Breen, R., Karlson, K. B., & Holm, A. (2013). Total, direct, and Indirect Effects in Logit and Probit Models. *Sociological Methods and Research*, *42*(2), 10.1177/0049124113494572.

[CR6] Brief, A. P., & Weiss, H. M. (2002). Organisational behavior: affect in the workplace. *Annual Review of Psychology*, *53*, 10.1146/annurev.psych.53.100901.135156. 10.1146/annurev.psych.53.100901.135156 11752487

[CR7] Cernas-Ortiz, D. A., & Wai-Kwan, L. (2021). Social connectedness and job satisfaction in mexican teleworkers during the pandemic: the mediating role of affective well-being. *Estudios Gerenciales*, *37*(158), 10.18046/j.estger.2021.158.4322.

[CR8] Chung, H. (2017). *Work autonomy, flexibility and Work-Life Balance*. University of Kent.

[CR10] Chung, H., Birkett, H., Forbes, S., & Seo, H. (2021). Covid-19, flexible Working, and implications for gender Equality in the United Kingdom. *Gender and Society*, *35*(2), 10.1177/08912432211001304.

[CR11] Chung, H., & Booker, C. (2022). Flexible Working and the Division of Housework and Childcare: Examining Divisions across Arrangement and Occupational Lines. Work, Employment and Society. 10.1177/09500170221096586

[CR12] Chung, H., & van der Horst, M. (2018). Women’s employment patterns after childbirth and the perceived access to and use of flexitime and teleworking. *Human Relations*, *71*(1), 10.1177/0018726717713828. 10.1177/0018726717713828 PMC5714156 29276304

[CR13] Chung H, van der Horst M (2020). Flexible working and unpaid overtime in the UK: the role of gender, parental and occupational status [Article]. Social Indicators Research.

[CR14] Clawson, D. (2014). Unequal time: gender, class, and family in employment schedules (N. Gerstel, Ed.) [Book]. Russell Sage Foundation.

[CR15] Cornwell, B. (2013). Switching Dynamics and the stress process. *Social Psychology Quarterly*, *76*(2), 10.1177/0190272513482133. 10.1177/0190272513482133 PMC4126261 25110381

[CR16] Coron C (2022). The gender-job satisfaction debate in the light of the “gendered organisations.”. Revue de Gestion Des Ressources Humaines.

[CR17] Craig, L., & Brown, J. E. (2017). Feeling rushed: gendered time quality, work hours, Nonstandard Work Schedules, and spousal crossover. *Journal of Marriage and Family*, *79*(1), 10.1111/jomf.12320.

[CR18] Desrochers, S., & Sargent, L. D. (2004). Boundary/Border theory and work-family integration 1. *Organization Management Journal*, *1*(1), 10.1057/omj.2004.11.

[CR19] England, P., Levine, A., & Mishel, E. (2020). Progress toward gender equality in the United States has slowed or stalled. *Proceedings of the National Academy of Sciences of the United States of America*, *117*(13), 10.1073/pnas.1918891117. 10.1073/pnas.1918891117 PMC7132302 32229559

[CR20] Erikson, R., & Goldthorpe, J. H. (2010). Has social mobility in britain decreased? Reconciling divergent findings on income and class mobility. *British Journal of Sociology*, *61*(2), 10.1111/j.1468-4446.2010.01310.x. 10.1111/j.1468-4446.2010.01310.x 20579052

[CR21] Fisher, C. D. (2002). Antecedents and consequences of real-time affective reactions at work. *Motivation and Emotion*, *26*(1), 10.1023/A:1015190007468.

[CR22] Fonner, K. L., & Roloff, M. E. (2010). Why teleworkers are more satisfied with their jobs than are office-based workers: when less contact is beneficial. *Journal of Applied Communication Research*, *38*(4), 10.1080/00909882.2010.513998.

[CR23] Glavin, P., & Schieman, S. (2012). Work-family role blurring and work-family conflict: the moderating influence of job resources and job demands. *Work and Occupations*, *39*(1), 10.1177/0730888411406295.

[CR24] Grönlund, A. (2007). More control, less conflict? Job demand-control, gender and work-family conflict. *Gender Work and Organization*, *14*(5), 10.1111/j.1468-0432.2007.00361.x.

[CR25] Hoang TTA, Knabe A (2020).

[CR26] Hofmans, J., Gelens, J., & Theuns, P. (2014). Enjoyment as a mediator in the relationship between task characteristics and work effort: an experience sampling study. *European Journal of Work and Organizational Psychology*, *23*(5), 10.1080/1359432X.2013.792229.

[CR27] Inanc H (2018). Unemployment, Temporary Work, and Subjective Well-Being: the Gendered Effect of Spousal Labor Market Insecurity. American Sociological Review.

[CR28] Judge TA, Bono JE, Thoresen CJ, Patton GK (2001). The job satisfaction-job performance relationship: a qualitative and quantitative review [Article]. Psychological Bulletin.

[CR29] Kan, M. Y., & Pudney, S. (2008). Measurement error in stylised and diary data on time use. *Sociological Methodology*, *38*(1), 10.1111/j.1467-9531.2008.00197.x.

[CR30] Kan MY, Laurie H (2018). Who is doing the Housework in Multicultural Britain? [Article]. Sociology (Oxford).

[CR31] Karasek, R. A. (1979). Job demands, job decision latitude, and Mental strain: implications for job redesign. *Administrative Science Quarterly*, *24*(2), 10.2307/2392498.

[CR32] Kim, H., Kim, Y., & Kim, D. L. (2019). Negative work–family/family–work spillover and demand for flexible work arrangements: the moderating roles of parenthood and gender. *International Journal of Human Resource Management*, *30*(3), 10.1080/09585192.2016.1278252.

[CR33] Kohler, U., Karlson, K. B., & Holm, A. (2011). Comparing coefficients of nested nonlinear probability models. *Stata Journal*, *11*(3), 10.1177/1536867x1101100306.

[CR34] Lee, M., & Jang, K. S. (2020). Nurses’ emotions, emotional labor, and job satisfaction. *International Journal of Workplace Health Management*, *13*(1), 10.1108/IJWHM-01-2019-0012.

[CR35] Li, L. Z., & Wang, S. (2020). Prevalence and predictors of general psychiatric disorders and loneliness during COVID-19 in the United Kingdom. *Psychiatry Research*, 291. 10.1016/j.psychres.2020.113267. 10.1016/j.psychres.2020.113267 PMC7326403 32623266

[CR36] Li LZ, Wang S (2022).

[CR37] Lopes H, Lagoa S, Calapez T (2014). Work autonomy, work pressure, and job satisfaction: an analysis of European Union countries [Article]. The Economic and Labour Relations Review: ELRR.

[CR38] Morganson, V. J., Major, D. A., Oborn, K. L., Verive, J. M., & Heelan, M. P. (2010). Comparing telework locations and traditional work arrangements. *Journal of Managerial Psychology*, *25*(6), 10.1108/02683941011056941.

[CR39] Offer, S., & Schneider, B. (2011). Revisiting the gender gap in time-use patterns: Multitasking and well-being among mothers and fathers in dual-earner families. *American Sociological Review*, *76*(6), 10.1177/0003122411425170.

[CR40] Pataki-Bittó, F., & Kun, Á. (2022). Exploring differences in the subjective well-being of teleworkers prior to and during the pandemic. *International Journal of Workplace Health Management*, *15*(3), 10.1108/IJWHM-12-2020-0207.

[CR41] Powell, A., & Craig, L. (2015). Gender differences in working at home and time use patterns: evidence from Australia. *Work Employment and Society*, *29*(4), 10.1177/0950017014568140.

[CR42] Sanz-Vergel, A. I., & Rodríguez-Muñoz, A. (2013). The spillover and crossover of daily work enjoyment and well-being: a diary study among working couples. *Revista de Psicologia Del Trabajo y de Las Organizaciones*, *29*(3), 10.5093/tr2013a24.

[CR43] Schieman, S., Whitestone, Y. K., & van Gundy, K. (2006). The nature of work and the stress of higher status. *Journal of Health and Social Behavior*, *47*(3), 10.1177/002214650604700304. 10.1177/002214650604700304 17066775

[CR44] Schweitzer, L., Lyons, S., Kuron, L. K. J., & Ng, E. S. W. (2014). The gender gap in pre-career salary expectations: a test of five explanations. *Career Development International*, *19*(4), 10.1108/CDI-12-2013-0161.

[CR45] Schweitzer, L., Ng, E., Lyons, S., & Kuron, L. (2011). Exploring the career pipeline: gender differences in pre-career expectations. *Relations Industrielles*, *66*(3), 10.7202/1006346ar.

[CR46] Song Y, Gao J (2019). Does Telework stress employees out? A study on working at Home and Subjective Well-Being for Wage/Salary workers [Article]. Journal of Happiness Studies.

[CR47] Teo, T. S., & Lim, V. K. (1998). Factorial dimensions and differential effects of gender on perceptions of teleworking. *Women in Management Review*, *13*(7), 10.1108/09649429810237105.

[CR48] Tietze, S., & Musson, G. (2010). Identity, identity work and the experience of working from home. *Journal of Management Development*, *29*(2), 10.1108/02621711011019288.

[CR49] Veenhoven, R., & Publications, G. (2008). *Sociological theories of Subjective Well-Being. The Science of Subjective Well-Being*. A Tribute to Ed Diener.

[CR50] Vega, R. P., Anderson, A. J., & Kaplan, S. A. (2015). A within-person examination of the Effects of Telework. *Journal of Business and Psychology*, *30*(2), 10.1007/s10869-014-9359-4.

[CR52] Wang, S., Li, L. Z., Zhang, J., & Rehkopf, D. H. (2021). Leisure time activities and biomarkers of chronic stress: the mediating roles of alcohol consumption and smoking. *Scandinavian Journal of Public Health*, *49*(8), 10.1177/1403494820987461. 10.1177/1403494820987461 33570003

[CR53] Wang S, Ling W, Lu Z, Wei Y, Li M, Gao L (2022). Can volunteering buffer the negative impacts of unemployment and economic inactivity on Mental Health? Longitudinal evidence from the United Kingdom. International Journal of Environmental Research and Public Health.

[CR54] Wang S, Lu Z (2022). Is Paid Inflexible Work Better than unpaid housework for women’s Mental Health? The moderating role of parenthood. Applied Research in Quality of Life.

[CR55] Wang, S., Zixin, L., Zhuofei, L., Shuanglong, L., & Rehkopf, D. (2022b). Work schedule control and allostatic load biomarkers: disparities between and within gender. Social Indicators Research, 0123456789. https://doi.org/10.1007/s11205-022-02940-7.

[CR56] Wegge, J., van Dick, R., Fisher, G. K., West, M. A., & Dawson, J. F. (2006a). A test of basic assumptions of affective events theory (AET) in call centre work. *British Journal of Management*, *17*(3), 10.1111/j.1467-8551.2006.00489.x.

[CR58] Weiss, H. M., Nicholas, J. P., & Daus, C. S. (1999). An examination of the Joint Effects of affective Experiences and Job beliefs on job satisfaction and variations in affective Experiences over Time. *Organisational Behavior and Human Decision Processes*, *78*(1), 10.1006/obhd.1999.2824. 10.1006/obhd.1999.2824 10092469

[CR59] West, C., & Zimmerman, D. H. (1987). Doing gender. *Gender & Society*, *1*(2), 10.1177/0891243287001002002.

[CR60] Wheatley, D. (2012). Good to be home? Time-use and satisfaction levels among home-based teleworkers. *New Technology Work and Employment*, *27*(3), 10.1111/j.1468-005X.2012.00289.x.

[CR61] Wheatley D (2017). Autonomy in Paid Work and Employee Subjective Well-Being [Article]. Work and Occupations.

[CR62] Williams JC, Blair-Loy M, Berdahl JL (2013). Cultural Schemas, Social Class, and the flexibility stigma [Article]. Journal of Social Issues.

[CR63] Wunder, C., & Heineck, G. (2013). Working time preferences, hours mismatch and well-being of couples: are there spillovers? *Labour Economics*, *24*, 10.1016/j.labeco.2013.09.002.

[CR64] Xue B, McMunn A (2021). Gender differences in unpaid care work and psychological distress in the UK Covid-19 lockdown [Article]. PloS One.

[CR65] Yucel, D., & Chung, H. (2021). Working from home, work–family conflict, and the role of gender and gender role attitudes. Community, Work and Family. 10.1080/13668803.2021.1993138

[CR66] Zuzanek J, Zuzanek T (2014). Of happiness and of despair, is there a measure? Time Use and Subjective Well-being [Article]. Journal of Happiness Studies.

